# Effects of trichostatin A on the intrinsic and extrinsic apoptotic pathway, cell viability, and apoptosis induction in hepatocellular carcinoma cell lines 

**Published:** 2021

**Authors:** Masumeh Sanaei, Fraidoon Kavoosi

**Affiliations:** *Research Center for Non-Communicable Diseases, Jahrom University of Medical Sciences, Jahrom, Iran *

**Keywords:** Trichostatin A, Extrinsic, Intrinsic, Pathway, Apoptosis

## Abstract

**Aim:**

The current study investigated the effect of trichostatin A (TSA) on mitochondrial/intrinsic [pro- (Bax, Bak, and Bim) and anti- (Bcl-2, Bcl-xL, and Mcl-1) apoptotic genes] and cytoplasmic/extrinsic (DR4, DR5, FAS, FAS-L, and TRAIL genes) pathways, histone deacetylase 1, 2, and 3, p53, p73, cell viability, and apoptosis in hepatocellular carcinoma (HCC) HCCLM3, MHCC97H, and MHCC97L cell lines.

**Background:**

Modulation of the acetylation status of histones, histones modification, plays an important role in regulating gene transcription and expression. Histone deacetylation controlled by histone deacetylases (HDACs) leads to gene downregulation. Histone deacetylase inhibitors (HDACIs) are an emerging class of therapeutics with potential anticancer effects. They can induce apoptosis by activating both extrinsic and intrinsic apoptotic pathways

**Methods:**

HCCLM3, MHCC97H, and MHCC97L cells were cultured and treated with TSA. To determine viability, apoptosis, and the relative expression level of the mentioned genes, MTT assay, cell apoptosis assay, and qRT-PCR, respectively, were conducted.

**Results:**

TSA up-regulated Bax, Bak, Bim, DR4, DR5, FAS, FAS-L, TRAIL, p53, and p73 and down-regulated Bcl-2, Bcl-xL, Mcl-1, histone deacetylases 1, 2, and 3 significantly, resulting in apoptosis induction. Maximal and minimal apoptosis was seen in the MHCC97H and HCCLM3 cell lines (93.94% and 39.68%, respectively) after 24 and 48 h. Therefore, the MHCC97H cell line was more sensitive to TSA.

**Conclusion:**

The current findings demonstrated that the HDAC inhibitor TSA can induce apoptosis and inhibit cell growth through both mitochondrial/intrinsic and cytoplasmic/extrinsic apoptotic pathways in hepatocellular carcinoma HCCLM3, MHCC97H, and MHCC97L cell lines.

## Introduction

 Modulation of the acetylation status of histones, histones modification, plays an important role in regulating gene transcription and expression. Increased histone acetylation increases gene transcription, whereas histone deacetylation induces gene silencing leading to gene downregulation. Histone acetylation is controlled by the opposing actions of two groups of enzymes, i.e. histone acetyltransferases (HATs) and histone deacetylases (HDACs). These two groups determine the pattern and status of the histone acetylation. The activity of HDACs has been linked to tumorigenesis (1). There are two mechanisms by which histone acetylation increases transcriptional activity. HATs transfer the acetyl moiety of acetyl coenzyme A by which the positive charge of the histone tails is neutralized and the structure of the chromatin is relaxed. This structure enables the transcriptional machinery to access the DNA and enhances gene transcription. Just the opposite, HDACs remove the acetyl group from the histone tails, leading to chromatin compaction and decreased gene transcription (2). In humans, there are 18 HDACs divided into three classes based on homology to yeast HDACs (3).

Histone deacetylase inhibitors (HDACIs) are an emerging class of therapeutic compounds with potential anticancer effects. They interfere with HDAC activity resulting in the regulation of biological events, such as cell differentiation, cell cycle, and apoptosis in cancer cells. These compounds bind to and inhibit HDAC enzymatic activity. To date, more than 50 naturally occurring or synthetic HDACIs have been developed. The biochemical structures of these drugs are extremely heterogeneous, from simple agents like valproate to more complicated compounds, such as MS-275. Crystallographic studies have indicated that the hydroxamic acid-based HDACIs suberoylanilide hydroxamic acid (SAHA) and trichostatin A (TSA) fit very well into the HDAC catalytic pocket (4). TSA was one of the first natural hydroxamate compounds found to inhibit HDAC activity, resulting in the reactivation of silenced tumor suppressor genes (TSGs) and apoptosis induction (5).

It has been reported that cell apoptosis is mediated by way of two molecular pathways leading to caspase activation, i.e. extrinsic death receptor and intrinsic mitochondrial pathways. The extrinsic pathway is initiated by ligations of transmembrane death receptors, whereas the intrinsic pathway requires disruption of the mitochondrial membrane to release cytochrome c. The B cell lymphoma-2 (BCL-2) family of proteins regulates the release of mitochondrial cytochrome c: BCL-2 and BCL-XL prevent the release of cytochrome c, whereas BAX promotes the release of cytochrome C (6). The BCL-2 family of proteins is the key regulator of apoptosis, or programmed cell death. They are located predominantly on the mitochondria, where the release of cytochrome c is regulated. Structurally, this family is divided into two major sub-groups, the anti-apoptotic proteins (e.g., BCL-2, BCL-XL, MCL-1, BFL-1, BCL-W, and BCL2L10) and the pro-apoptotic proteins (such as BAK, BAX, BOK, BIM, PUMA, NOXA, BID, etc.) (7). 

The extrinsic pathway is triggered by the binding of death ligands of the tumor necrosis factor (TNF) family, such as TNF-related apoptosis-inducing ligand (TRAIL or Apo2L), to their appropriate death receptors (DRs) on the cell surface (8). TRAIL is a type II transmembrane protein, which was originally identified based on sequence homology to Fas ligand (FasL) and TNF. It exerts its function by engaging its receptors expressed on the surface of target cells. To date, four human receptors specific for TRAIL have been reported, comprising TRAIL-R1/DR4, TRAIL-R2/DR5, TRAIL-R3/DcR1, and TRAIL-R4/DcR2 (9). As mentioned, the extrinsic pathway is initiated by the binding of death receptors, including tumor necrosis factor (TNF) receptor-1 (TNFR-1), Fas (Apo-1 or CD95), TNF-related apoptosis-inducing ligand (TRAIL orApo2-L) receptors (DR-4 and -5), and DR-3 (Apo3) and DR-6, to their ligands, such as TNF, FasL, TRAIL, and TL1A (Apo3L) (10, 11). 

HDACIs have been shown to upregulate the expression of both death receptors and their ligands in transformed cells (12, 13). In vitro studies have demonstrated that HDACIs induce apoptosis through activation of both extrinsic and intrinsic apoptotic pathways (14). It has been indicated that the histone deacetylase inhibitor TSA induces Bax-dependent apoptosis in colorectal cancer cell lines by both p53-dependent mechanisms (15). Moreover, p53 is essential for the apoptotic response to TSA and SAHA in ovarian cancer, which is associated with activation of caspase-9, caspase-2, caspase-8, and caspase-7 (16). Other researchers have shown that TSA induces apoptosis by restoring both p73 and Bax but not p53 expression (17). Additionally, it has been reported that TSA induces the cell apoptosis of NCI-H157 human lung cancer cells through a signaling cascade of Fas/FasL-mediated extrinsic and mitochondrial-mediated intrinsic caspases pathway (18). 

**Table 1:** IC50 values of TSA determined by MTT assay

Cell line	Duration/Hour	IC50/ μM	LogIC50	R squared
HCCLM3	24	3.273	0.5149	0.6560
HCCLM3	48	1.552	0.1908	0.9773
MHCC97H	24	2.589	0.4131	0.9024
MHCC97H	48	1.1908	0.2805	0.8181
MHCC97L	24	3.622	0.5589	0.8281
MHCC97L	48	1.908	0.2805	0.9821

The current study investigated the effects of TSA on mitochondrial/intrinsic [pro- (Bax, Bak, and Bim) and anti- (Bcl-2, Bcl-xL, and Mcl-1) apoptotic genes] and cytoplasmic/extrinsic (DR4, DR5, FAS, FAS-L, and TRAIL genes) pathways, histone deacetylase 1, 2, and 3, p53, p73, cell viability, and apoptosis in hepatocellular carcinoma (HCC) HCCLM3, MHCC97H, and MHCC97L cell lines. 

## Materials

**Materials**

Hepatocellular carcinoma HCCLM3, MHCC97H, and MHCC97L cell lines were purchased from the National Cell Bank of Iran-Pasteur Institute. TSA and Dulbecco’s modified Eagle’s medium (DMEM) were obtained from Sigma (St. Louis, MO, USA) and dissolved in dimethyl sulfoxide (DMSO) to make a working stock solution. Further concentrations of TSA were obtained by diluting the provided stock solution. Other necessary materials and kits were purchased as provided for previous works (19, 20). The HCCLM3, MHCC97H, and MHCC97L cells were maintained in DMEM supplemented with fetal bovine serum 10% and antibiotics in a humidified atmosphere of 5% CO2 in air at 37 ℃. This work is a lab trial study that was approved by the Ethics Committee of Jahrom University of Medical Science with the code number IR.JUMS.REC.1399.053. 

**Cell culture and cell viability**

HCCLM3, MHCC97H, and MHCC97L cells were cultured in DMEM supplemented with 10% FBS and antibiotics (100 U/mL streptomycin and 100 U/mL penicillin) at 37 °C in 5% CO2 for 24 h and then seeded into 96-well plates (3 × 10^5 ^cells per well). After 24 h, the medium was replaced with a medium containing TSA with various doses (0, 0.5, 1, 2.5, 5, and 10 μM). The control groups were treated with DMSO at a concentration of 0.05%. After 24 and 48 h, the cells, treated and untreated, were investigated by MTT assay according to standard protocols to determine cell viability. The MTT solution was added to each well for 4 h at 37 ℃, and then the MTT solution was changed by DMSO and shaken for 10 min to dissolve all of the crystals. The MTT assay, a quantitative colorimetric assay, is based on the living cell’s ability to reduce the tetrazolium salt MTT. The mitochondrial succinate-dehydrogenases of viable cells cleave the tetrazolium ring in active mitochondria into formazan crystals which can be dissolved in DMSO. Finally, the optical density was detected by a microplate reader at a wavelength of 570 nM. Each experiment was repeated three times.

**Cell apoptosis assay**

To determine HCCLM3, MHCC97H, and MHCC97L cell apoptosis, the cells were cultured at a density of 3 × 10^5^ cells/well and treated with TSA, based on the IC50 values indicated in Table 1, for 24 and 48 h. Then, both treated and untreated cells were harvested by trypsinization, washed with cold PBS, and resuspended in binding buffer (1x). Finally, Annexin-V-(FITC) and PI were used according to the protocol to determine the apoptotic cells by FACScan flow cytometry (Becton Dickinson, Heidelberg, Germany).

**Real-time Quantitative Reverse Transcription Polymerase Chain Reaction (qRT-PCR)**

To determine the relative expression levels of Bax, Bak, Bim, Bcl-2, Bcl-xL, Mcl-1, DR4, DR5, FAS, FAS-L, TRAIL, histone deacetylase inhibitors 1, 2, and 3, p53, and p73, qRT-PCR analyses were done. The HCCLM3, MHCC97H, and MHCC97L cells (at a density of 3 × 10^5^ cells/well) were treated with TSA, based on the IC50 values indicated in Table 1, for 24 and 48 h, except the control groups which were treated with DMSO only. Then qRT-PCR was done as in our previous works (21). The primer sequences are shown in Table 2 (22-33).

**Statistical analysis**

The database was set up with the SPSS 16.0 software package (SPSS Inc., Chicago, Illinois, USA) and Graph Pad Prism 8.0 for data analysis. Results are expressed as mean ± standard deviation (SD) for n=3 independent experiments. Statistical comparisons between groups were performed with ANOVA (oneway ANOVA) and the Tukey test. A *p*-value < 0.05 was considered as a significant difference. 

## Results

**Results of cell viability by the MTT assay**

**Figure 1:**
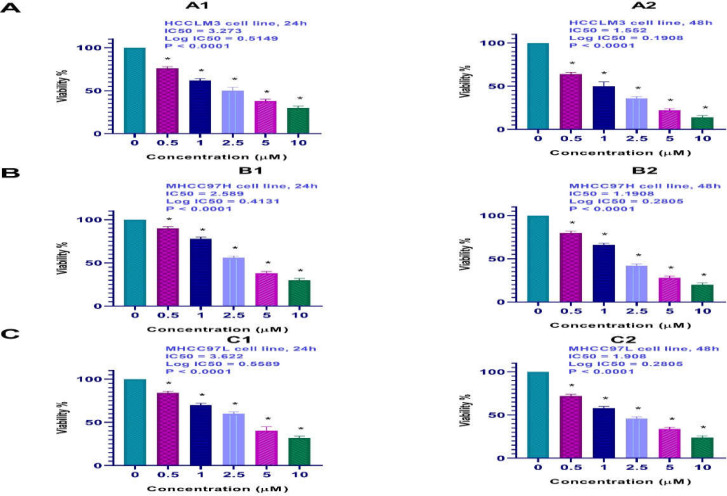
Effect of TSA on viability of HCCLM3, MHCC97H, and MHCC97L cells. The cells were treated with and without TSA, and cell viability was evaluated by MTT assay. Each experiment was conducted in triplicate. Mean values from the three experiments ± standard error of mean are shown. Asterisks indicate significant differences between treated and untreated cells

The viability of the HCCLM3, MHCC97H, and MHCC97L cells treated with various doses of TSA (0, 0.5, 1, 2.5, 5, and 10 μM) was investigated by MTT assay. As shown in Figure 1, TSA induced significant cell growth inhibition in a dose-dependent manner (*p*< 0.001). The IC50 value was calculated by Graph pad prism 8 as indicated in Table 1. 

**Table 2:** Primer sequences of Bax, Bak, Bim, Bcl-2, Bcl-xL, Mcl-1, DR4, DR5, FAS, FAS-L, TRAIL, histone deacetylase inhibitors 1, 2, and 3, p53, and p73 genes

Primer	Primer sequences (5' to 3')	Product length	Reference
Bax Forward Reverse	AGTAACATGGAGCTGCAGAGGAT GCTGCCACTCGGAAAAAGAC	77 bp	
Bak Forward Reverse	TGAAAAATGGCTTCGGGGCAAG CTCTCAAACGGCTGGTGGCAATC	367 bp	
Bim Forward Reverse	ATTACCAAGCAGCCGAAGAC TCCGCAAAGAACCTGTCAAT	101 bp	
Bcl-2 Forward Reverse	TGGCCAGGGTCAGAGTTAAA TGGCCTCTCTTGCGGAGTA	147 bp	
Bcl-xL Forward Reverse	TCCTTGTCTACGCTTTCCACG GGTCGCATTGTGGCCTTT	62 bp	
Mcl-1 Forward Reverse	AAAGCCTGTCTGCCAAAT CCTATAAACCCACCACTC	198 bp	
DR4 Forward Reverse	CAGAACATCCTGGAGCCTGTAAC ATGTCCATTGCCTGATTCTTTGTG	299 bp	
DR5 Forward Reverse	TGCAGCCGTAGTCTTGATTG GCACCAAGTCTGCAAAGTCA	389 bp	
FAS Forward Reverse	ATGCTGGGCATCTGGACCCT GCCATGTCCTTCATCACACAA	366 bp	
FAS-L Forward Reverse	TGGAATTGTCCTGCTTTCTGG TGTTGCAAGATTGACCCCG	113 bp	
TRAIL Forward Reverse	ACCAACGAGCTGAAGCAGAT TCCTTGATGATTCCCAGGAG	213 bp	
HDAC1 Forward Reverse	AACCTGCCTATGCTGATGCT CAGGCAATTCGTTTGTCAGA	374 bp	
HDAC2 Forward Reverse	GGGAATACTTTCCTGGCACA ACGGATTGTGTAGCCACCTC	314 bp	
HDAC3 Forward Reverse	TGGCTTCTGCTATGTCAACG GCACGTGGGTTGGTAGAAGT	328 bp	
P53 Forward Reverse	CAGCCAAGTCTGTGACTTGCACGTAC CTATGTCGAAAAGTGTTTCTGTCATC	292 bp	
P73 Forward Reverse	AACGCTGCCCCAACCACGAG GCCGGTTCATGCCCCCTACA	231 bp	
GAPDH Forward Reverse	GAAGGTGAAGGTCGGAGTC GAAGATGGTGATGGGATTTC	172 bp	

**Results of determination of cell apoptosis**

**Figure 2:**
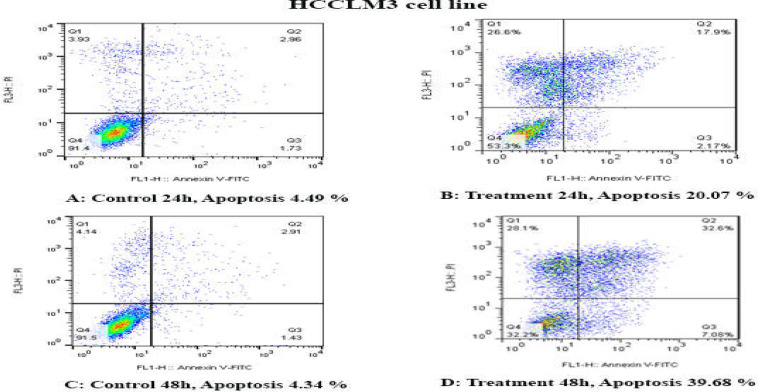
The apoptosis-inducing effect of TSA was investigated by flow cytometric analysis of HCCLM3 cells stained with Annexin V and propidium iodide. The results indicated that TSA significantly induced cell apoptosis after 24 and 48 h of treatment

**Figure 3:**
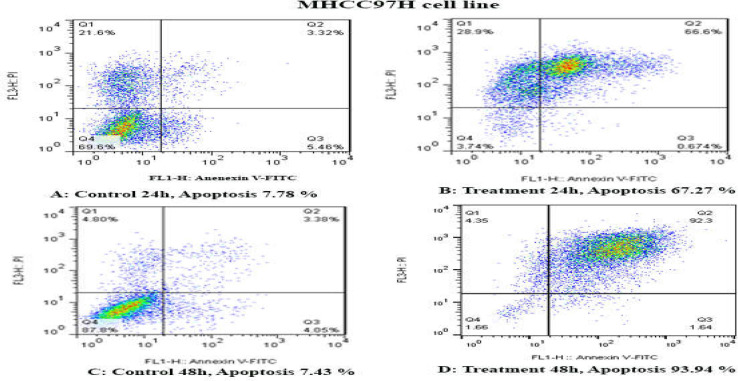
The apoptosis-inducing effect of TSA was investigated by flow cytometric analysis of MHCC97H cells stained with Annexin V and propidium iodide. The results indicated that TSA significantly induced cell apoptosis after 24 and 48 h of treatment

**Figure 4:**
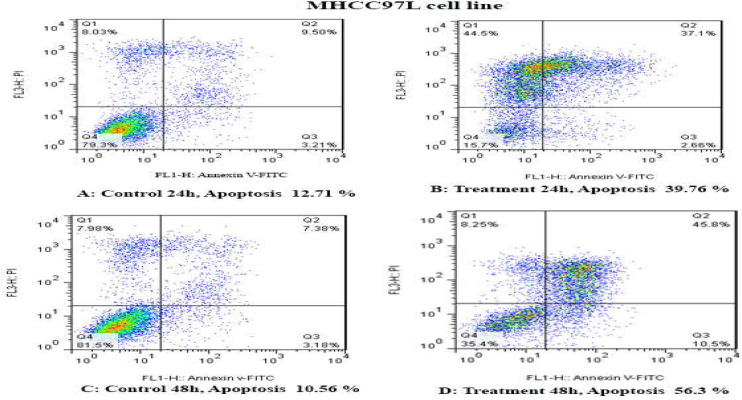
The apoptosis-inducing effect of TSA was investigated by flow cytometric analysis of MHCC97L cells stained with Annexin V and propidium iodide. The results indicated that TSA significantly induced cell apoptosis after 24 and 48 h of treatment

**Figure 5:**
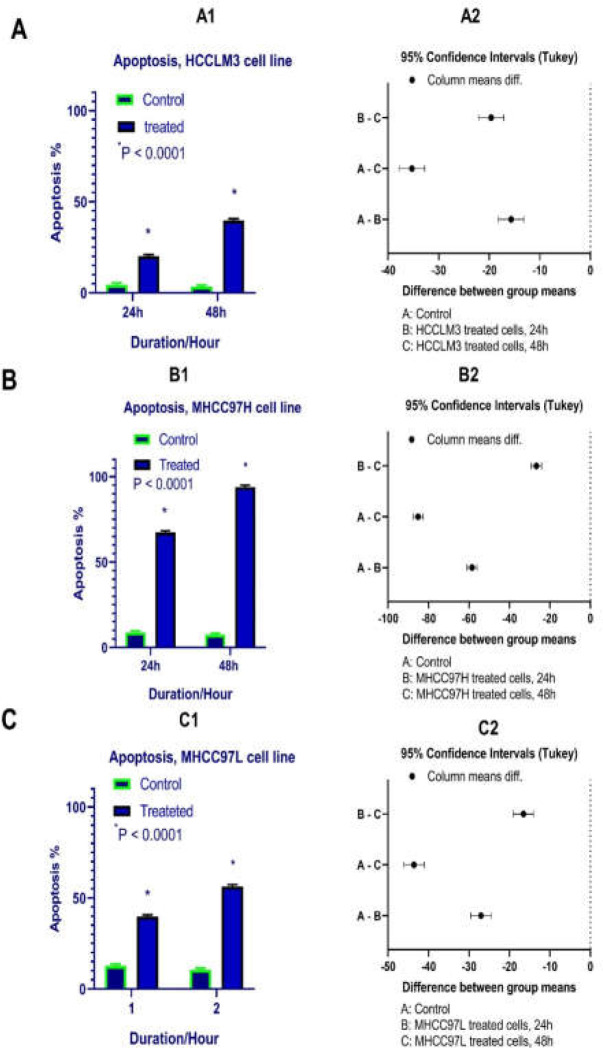
Apoptotic effects of TSA on HCCLM3, MHCC97H, and MHCC97L cells versus control groups at different periods (24 and 48h). Results were obtained from three independent experiments and expressed as mean ± standard error. Statistical analysis indicated significant differences between treated and untreated cells (A2, B2, and C2).

**Figure 6:**
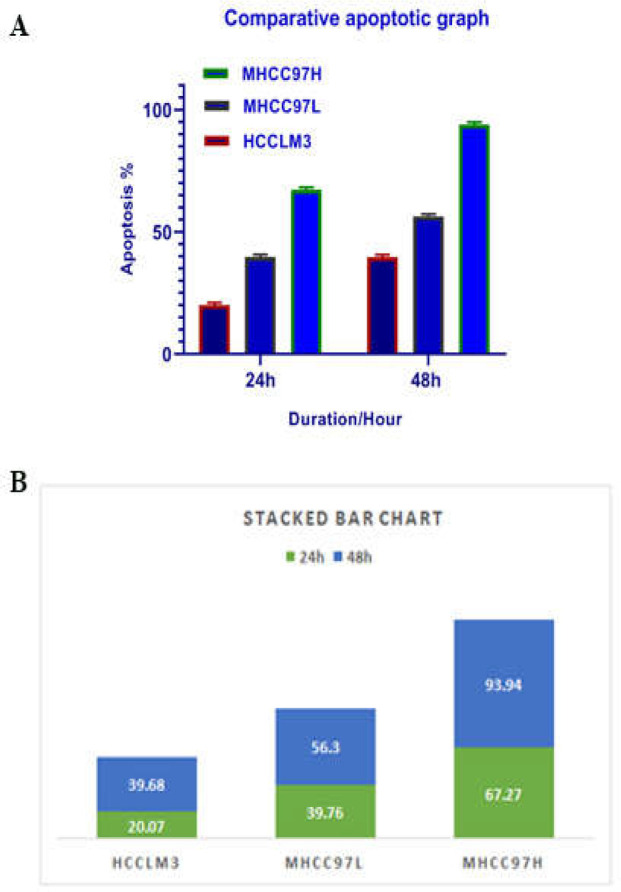
Comparative analysis of the effects of TSA on HCCLM3, MHCC97H, and MHCC97L cells (A). Maximal and minimal apoptosis was seen in the MHCC97H and HCCLM3 cell lines, respectively, after 24 and 48 h (B).

To determine cell apoptosis, HCCLM3, MHCC97H, and MHCC97L cells were treated with TSA, based on IC50 values, for 24 and 48 h and then stained using annexin-V-(FITC) and PI to determine apoptotic cells in the early and late stages of apoptosis. As indicated in Figures 2-6, TSA induced significant cell apoptosis in all three cell lines (*p*<0.001).

**Results of determining gene expression in HCCLM3 cell line**

**Figure 7:**
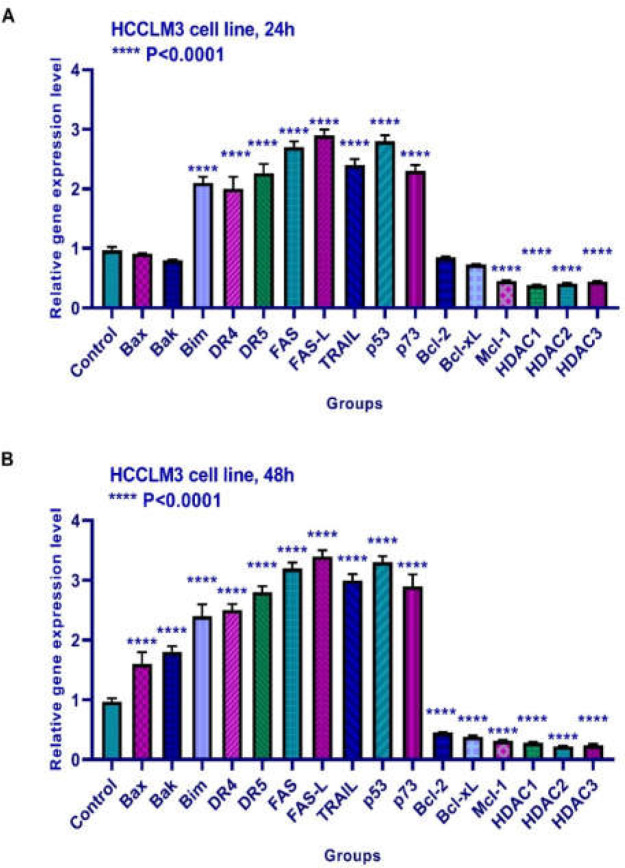
Relative expression levels of Bax, Bak, Bim, Bcl-2, Bcl-xL, Mcl-1, DR4, DR5, FAS, FAS-L, TRAIL, histone deacetylase inhibitors 1, 2, and 3, p53, and p73 in HCCLM3 cells treated with TSA for 24 and 48 h. Quantitative reverse transcription-polymerase chain reaction analysis demonstrated that this compound up-regulated Bim, DR4, DR5, FAS, FAS-L, TRAIL, p53, and p73 and down-regulated Mcl-1, histone deacetylases 1, 2, and 3 significantly after 24 h of treatment. It had no significant effect on the expression of Bax, Bak, Bcl-2, and Bcl-xL after 24 h. Furthermore, this compound up-regulated Bax, Bak, Bim, DR4, DR5, FAS, FAS-L, TRAIL, p53, and p73 and down-regulated Bcl-2, Bcl-xL, Mcl-1, and histone deacetylases 1, 2, and 3 significantly after 48 h of treatment. Asterisks indicate significant differences between treated cells and the control group. Data is presented as means ± standard error. ^****^p<0.0001

The effects of TSA on Bax, Bak, Bim, Bcl-2, Bcl-xL, Mcl-1, DR4, DR5, FAS, FAS-L, TRAIL, histone deacetylases 1, 2, and 3, p53, and p73 were evaluated by quantitative real-time RT-PCR analysis. The result of quantitative reverse transcription-polymerase chain reaction analysis demonstrated that this compound up-regulated Bim, DR4, DR5, FAS, FAS-L, TRAIL, p53, and p73 and down-regulated Mcl-1, histone deacetylases 1, 2, and 3 significantly after 24 h of treatment. It had no significant effect on the expression of Bax, Bak, Bcl-2, or Bcl-xL after 24 h. Furthermore, this compound up-regulated Bax, Bak, Bim, DR4, DR5, FAS, FAS-L, TRAIL, p53, and p73 and down-regulated Bcl-2, Bcl-xL, Mcl-1, and histone deacetylases 1, 2, and 3 significantly after 48 h of treatment (Figure 7).

**Results of determining gene expression in MHCC97H cell line**

**Figure 8:**
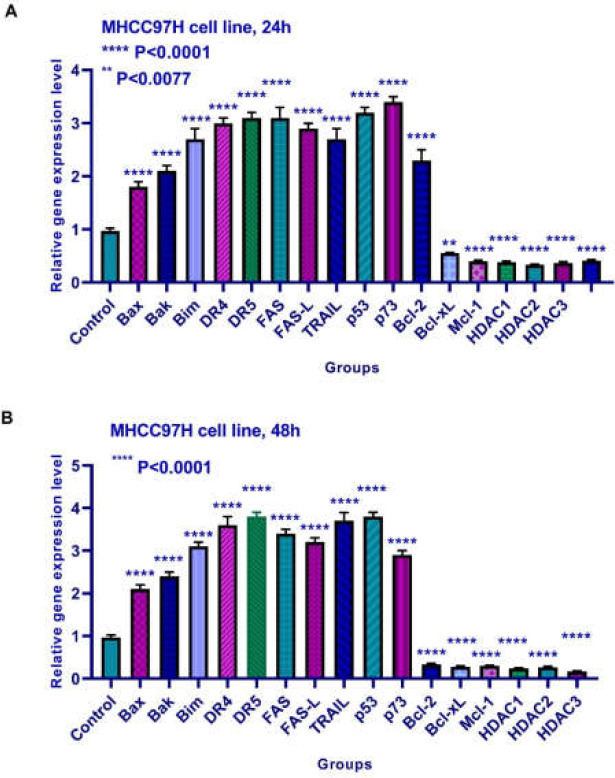
Relative expression levels of Bax, Bak, Bim, Bcl-2, Bcl-xL, Mcl-1, DR4, DR5, FAS, FAS-L, TRAIL, histone deacetylase inhibitors 1, 2, and 3, p53, and p73 in MHCC97H cells treated with TSA for 24 and 48 h. Quantitative reverse transcription-polymerase chain reaction analysis demonstrated that this compound up-regulated Bax, Bak, Bim, DR4, DR5, FAS, FAS-L, TRAIL, p53, and p73 and down-regulated Bcl-2, Bcl-xL, Mcl-1, and histone deacetylases 1, 2, and 3 significantly after 24 and 48 h of treatment. Asterisks indicate significant differences between treated cells and the control group. Data is presented as means ± standard error. ****p and **p <0.0001 and p <0.0077, respectively

The effects of TSA on the Bax, Bak, Bim, Bcl-2, Bcl-xL, Mcl-1, DR4, DR5, FAS, FAS-L, TRAIL, histone deacetylases 1, 2, and 3, p53, and p73 were evaluated by quantitative real-time RT-PCR analysis. Quantitative reverse transcription-polymerase chain reaction analysis demonstrated that this compound up-regulated Bax, Bak, Bim, DR4, DR5, FAS, FAS-L, TRAIL, p53, and p73 and down-regulated Bcl-2, Bcl-xL, Mcl-1, and histone deacetylases 1, 2, and 3 significantly after 24 and 48 h of treatment (Figure 8). 

**Results of determining gene expression in MHCC97L cell**

**Figure 9:**
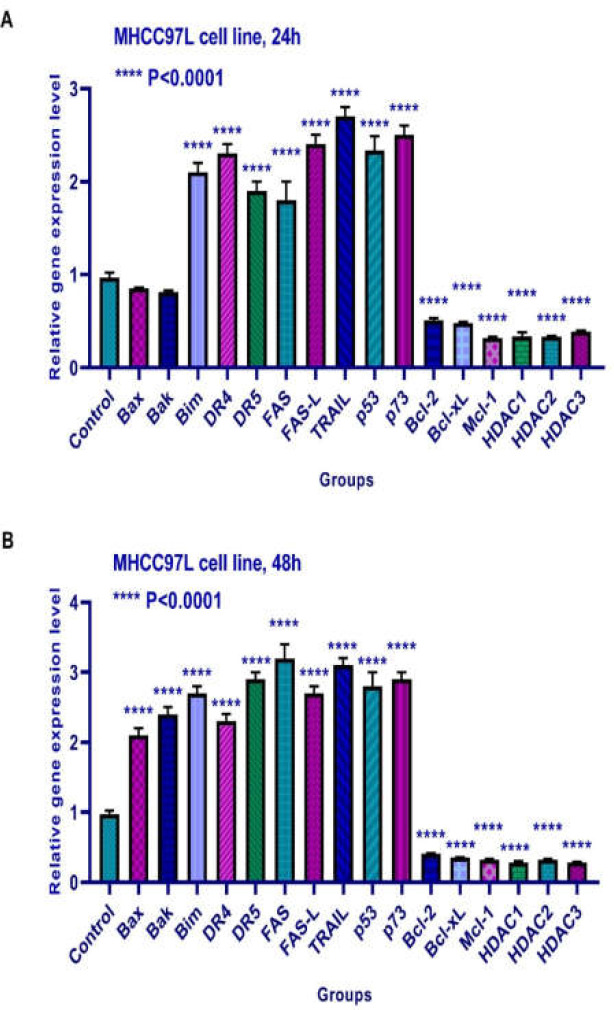
Relative expression levels of Bax, Bak, Bim, Bcl-2, Bcl-xL, Mcl-1, DR4, DR5, FAS, FAS-L, TRAIL, histone deacetylase inhibitors 1, 2, and 3, p53, and p73 in MHCC97L cells treated with TSA for 24 and 48 h. Quantitative reverse transcription-polymerase chain reaction analysis demonstrated that this compound up-regulated Bim, DR4, DR5, FAS, FAS-L, TRAIL, p53, and p73 and down-regulated Bcl-2, and Bcl-xL, Mcl-1, and histone deacetylases 1, 2, and 3 significantly after 24 h of treatment. It had no significant effect on the expression of Bax and Bak after 24 h. Furthermore, this compound up-regulated Bax, Bak, Bim, DR4, DR5, FAS, FAS-L, TRAIL, p53, and p73 and down-regulated Bcl-2, Bcl-xL, Mcl-1, and histone deacetylases 1, 2, and 3 significantly after 48 h of treatment. Asterisks indicate significant differences between treated cells and the control group. Data is presented as means ± standard error. p <0.0001

The effects of TSA on Bax, Bak, Bim, Bcl-2, Bcl-xL, Mcl-1, DR4, DR5, FAS, FAS-L, TRAIL, and histone deacetylases 1, 2, and 3, p53, and p73 were evaluated by quantitative real-time RT-PCR analysis. Quantitative reverse transcription-polymerase chain reaction analysis demonstrated that this compound up-regulated Bim, DR4, DR5, FAS, FAS-L, TRAIL, p53, and p73 and down-regulated Bcl-2, and Bcl-xL, Mcl-1, and histone deacetylases 1, 2, and 3 significantly after 24 h of treatment. It had no significant effect on the expression of Bax and Bak after 24 h. Furthermore, this compound up-regulated Bax, Bak, Bim, DR4, DR5, FAS, FAS-L, TRAIL, p53, and p73 and down-regulated Bcl-2, Bcl-xL, Mcl-1, and histone deacetylases 1, 2, and 3 significantly after 48 h of treatment (Figure 9).

## Discussion

Histone deacetylase (HDAC) inhibitors can induce apoptosis through both intrinsic and extrinsic pathways (34). It has been reported that they activate the intrinsic pathway through the upregulation of several pro-apoptotic genes such as Bim, Bid, and Bmf. They engage the extrinsic pathway through the upregulation of DR expression, upregulation of ligands such as TRAIL, and reductions in c-FLIP (35). In vitro studies have demonstrated that histone deacetylase inhibitors induce apoptosis through the signaling cascade of Fas/FasL-mediated extrinsic and mitochondrial-mediated intrinsic caspases pathway (36). 

The current findings demonstrated that TSA up-regulated Bax, Bak, Bim, DR4, DR5, FAS, FAS-L, TRAIL, p53, and p73 and down-regulated Bcl-2, Bcl-xL, Mcl-1, and histone deacetylases 1, 2, and 3 significantly, resulting in apoptosis induction. 

Similarly, our previous work indicated that TSA can induce apoptosis by inhibiting deacetylases 1, 2, and 3 gene expression and up-regulating p21, p27, and p57 in the breast cancer SK-BR-3 cell line (37). In leukemia cell lines (Jurkat, HL60, K562, and U937), TSA has been shown to induce apoptosis through multiple mechanisms, including the up-regulation of DR4, DR5, Bax, Bak, Bim, PUMA, and Noxa, down-regulation of Mcl-1, Bcl-XL, Bcl-2, and cFLIP, the release of mitochondrial proteins (cytochrome c), induction of p21WAF1/CIP1 and p27KIP1, and activation of caspase-3 (38). Additionally, HDACIs can induce apoptosis in chronic lymphocytic leukemia (CLL) through the inactivation of Bcl-2 family members by increases in Noxa and Bim (39). Other researchers have demonstrated that TSA and valproic acid (VPA) increase the pro-apoptotic Bim level and reduce the anti-apoptotic Mcl-1 level in pancreatic cancer (Panc1 and PaCa44) cells (40). 

Moreover, HDACIs increase the activation of caspases and Bid and the inactivation of the anti-apoptotic proteins Bcl-x, XIAP, RIP, and survivin, thereby increasing the pro- to anti-apoptotic protein ratio (41). In colorectal cancer (HCT116 and HT29) cell lines, TSA induces the expression of Bax and decreases the expression of Bcl-2 and Bcl-xL (42). 

Many in vitro experiments have proven that these compounds inﬂuence DR5 (death receptor 5), death receptors TRAIL (TNF-related apoptosis-inducing ligand), TNF (tumor necrosis factor), Fas (TNF superfamily 6), TNF-related ligands Fas-L, TLA1 (transparent leaf area peptide), and LIGHT (TNF superfamily member 14). It can be concluded that in cancer cells exposed to HDACIs, pro-apoptotic genes involved in the intrinsic (BAK, BAX, and APAF1) and/or extrinsic (FAS, FAS-L, TRAIL, DR5, and TNF-α) apoptotic pathways are up-regulated, while anti-apoptotic genes (XIAP, Bcl-2) are downregulated (43). 

The results of the current study indicated that TSA up-regulated p53 and p73 expression significantly. Similar to the current results, it has been reported that TSA induces apoptosis by p53 up-regulation and Bcl-2 down-regulation in the HCC HepG2 cell line (44). Inconsistent with these findings, TSA has been shown to induce apoptosis through Bax up-regulation in human gastric cell lines (45). Similarly, TSA enhances the apoptosis of cervical cancer cells through the overexpression of p53 and p73 (46). 

In the present study, TSA had no significant effect on the expression of Bax, Bak, Bcl-2, or Bcl-xL in the HCCLM3 cell line, nor on the expression of Bax and Bak in the MHCC97L cell line after 24 h of treatment. No report on the effects of TSA on the expression of Bax, Bak, Bcl-2, and Bcl-xL in HCCLM3, MHCC97H, and MHCC97L cell lines was found in a literature search. It may be possible to change the expression of the mentioned genes with high concentrations of TSA. Therefore, the evaluation of a high dose of TSA on these cell lines is recommended.

The current findings demonstrate that the HDAC inhibitor TSA can induce apoptosis and inhibit cell growth through both mitochondrial/intrinsic and cytoplasmic/extrinsic apoptotic pathways in hepatocellular carcinoma HCCLM3, MHCC97H, and MHCC97L cell lines.
